# Study on Mechanical and Microstructural Evolution of P92 Pipes During Long-Time Operation

**DOI:** 10.3390/ma17205092

**Published:** 2024-10-18

**Authors:** Liying Tang, Zheyi Yang, Xionghua Cui, Lei Zhang, Jiang Li

**Affiliations:** Xi’an Thermal Power Research Institute Co., Ltd., Xi’an 710054, China; tangliying@tpri.com.cn (L.T.); cuixionghua@tpri.com.cn (X.C.); zhanglei@tpri.com.cn (L.Z.); lijiang@tpri.com.cn (J.L.)

**Keywords:** P92, USC, long-term service, creep life consumption, mechanical property, microstructure evolution

## Abstract

To investigate the mechanical properties and microstructure evolution of P92 steel during long-term service, the operated P92 main steam pipes from the first ultra-supercritical units in China were sectioned into samples representing various service durations and stresses (0# (as-received state, 1# (82,000 h, 67.3 MPa), 2# (85,000 h, 78.0 MPa), and 3# (100,000 h, 80.3 MPa)). Thereafter, a comprehensive assessment of their mechanical properties, including tensile strength, impact, hardness, and creep resistance, as well as a detailed microstructure analysis, was carried out. The effect of stress on the aging of material properties during operation is discussed. The results show that the circumferential stress caused by the increase in the internal steam pressure can significantly promote the creep life consumption of P92 steel, resulting in the degradation of mechanical properties and the expedited aging of the microstructure. The *R*_p0.2_ and *R*_m_ of the P92 main steam pipe at room temperature and 605 °C decreased with the service time increase, reflecting the influence of stress in operation, which is expected to be used for the residual life evaluation of P92 steel. The relationship between the impact absorption energy (FATT50), Brinell hardness, and the operating time of P92 operating pipes is non-monotonic, indicating that these parameters are not sensitive indicators of material aging due to stress. The evaluation of performance degradation in P92 operating pipes due to stress-induced aging is not reliably discernible through optical metallography alone. To achieve a thorough assessment, the use of transmission electron microscopy (TEM) is essential.

## 1. Introduction

P92 steel is a typical 9% chromium ferritic steel, belonging to the improved Cr-Mo steel series, and is widely used in ultra-supercritical (USC) power station boilers and steam pipelines, among other high-temperature and high-pressure applications [1]. It possesses excellent high-temperature strength, creep resistance, and oxidation resistance, enabling it to operate stably over long periods in environments exceeding 600 °C [2]. The primary alloying elements in P92 steel are chromium, molybdenum, and tungsten, with additional trace elements such as vanadium, nitrogen, and boron to further enhance its high-temperature creep resistance and corrosion resistance [3]. The initial microstructure of the material, as observed under STEM imaging, reveals slender, thin martensitic plates arranged almost parallel to each other. The layered microstructure consists of austenite grains, blocky, packet, and martensitic plates [4,5]. Moreover, elliptical M_23_C_6_ and fine MX carbide particles are distributed along the grain boundaries and within the grains [6]. In addition to these complex structures and dispersed precipitates, a high dislocation density is another significant reason for the excellent fatigue and creep resistance of 9% chromium steel [7]. Furthermore, P92 steel also exhibits excellent structural stability and weldability, making it more reliable during manufacturing and maintenance processes. Its oxidation and corrosion resistance at high temperatures effectively delay material aging and oxidation, extending its service life [8]. The impact resistance of P92 steel has also been enhanced, allowing it to maintain good structural integrity under harsh operating conditions [3]. These characteristics have made P92 steel widely used in the energy, petrochemical, and power industries. Therefore, since the commissioning of China’s first USC unit at the Huaneng Yuhuan Power Plant in 2006, there has been a widespread deployment of 600 °C-class USC power generating units. These units are characterized by the incorporation of essential thick-walled components, notably a high-temperature superheater header and main steam piping, which are constructed from P92 steel.

The exceptional mechanical properties of P92 steel are primarily attributed to its tempered martensitic structure [9,10,11]. The mechanical properties of the material are substantially influenced by the combined effects of solution strengthening, precipitation strengthening, and dislocation strengthening, with lath strengthening emerging as the most pivotal factor. X. Jin found that the martensitic lath contributes over 60% of the theoretical tensile strength during service [12,13]. Furthermore, the finer the lath size, the more effective the strengthening [14]. M_23_C_6_ particles predominantly precipitate at the austenite grain boundaries, while fine MX carbonitrides are uniformly distributed along the lath boundaries. The coarsening of martensite laths and martensite recovery, the formation of Laves and Z phase, and the coarsening of Laves phase and M_23_C_6_ precipitates would take place during high-temperature long-term exposure, creep, or aging [15]. The rapid coarsening and clustering of the Laves phase have been identified as primary factors in the reduction of T/P92 steel’s creep strength and toughness [16,17]. Concurrently, the modified Z-phase acts as a diagnostic marker for the aging process of the material.

Numerous studies have employed aging treatments at temperatures higher than the operational service temperatures to accelerate the aging process of material structures and properties. Research findings have indicated that aging temperature significantly influences the room temperature strength, high-temperature strength, hardness, and microstructural evolution of P92 steel [18,19,20]. Although the mechanical properties of P92 steel exhibit a decline within the first 1000 h of aging at temperatures of 650 °C and 680 °C, distinct evolutionary trends become apparent with extended aging durations at 650 °C; the properties stabilize after 29,000 h, whereas at 680 °C, they continue to decline even after 18,000 h. High-temperature aging tests, although economical, deviate from real service conditions due to their inability to precisely mimic the thermodynamic stability of precipitated phases, which is temperature-dependent. For instance, the Laves phase, a critical factor in P92 steel performance, precipitates at around 700 °C. Accelerated aging above this temperature can result in significant deviations in the types, sizes, and quantities of precipitated phases when compared to those under actual service conditions. Moreover, aging treatments do not account for the impact of stress on material performance and microstructure [21]. Studies [22] have demonstrated that microstructural evolution and the corresponding performance under stress-free aging cannot represent the true conditions experienced under stress during service. However, due to the constraints of test costs and duration, these studies often resort to increasing temperature or stress to expedite the testing process. The selected stresses are significantly higher than actual service stresses, rendering the implications of these stress levels ambiguous. Additionally, some studies have evaluated material performance and analyzed the microstructure of long-serving pipe sections sampled from operational environments, revealing significant degradation compared to the original pipes [23,24]. Properties such as tensile strength, plasticity, impact toughness, and hardness have shown marked declines. Nonetheless, due to the challenges in sampling thick-walled pipes, the available data are sparse and lack systematic analysis.

As the most extensively utilized material for thick-walled components in USC units, understanding the changes in microstructural and mechanical properties of P92 steel over its service life is critical for ensuring the safety and reliability of these systems. Due to the suboptimal long-term creep rupture data of P92 steel, the ASME standard has undergone two revisions to decrease the permissible stress values for P92 steel, with adjustments made in 2006 and subsequently in 2023. The first batch of USC units in China was constructed using the allowable stress values from the ASME CC2179-1999 standard [25]. Compared to this, the allowable stress at 610 °C in the 2023 version has decreased by 16%. Even though the stress on the main steam pipelines of the first batch of USC units is slightly higher than the allowable stress, it remains considerably lower than the stress levels used in laboratory creep rupture tests. The alterations in microstructure and mechanical properties that occur throughout the service of these pipelines are thus of significant reference value. This paper provides an extensive comparative analysis of the main steam pipelines from China’s first batch of USC units, which have accumulated operational durations of 82,000 h, 85,000 h, and 100,000 h, respectively, contrasted with pristine samples in the as-received (0 h) condition. This research delves into the progression of mechanical properties and microstructures during long-term service and examines the impact of stress on the microstructural and mechanical properties of the material.

## 2. Test Materials and Methods

The test material analyzed in this study originated from the main steam pipeline of China’s inaugural batch of USC units, designed to operate at a temperature of 610 °C, with a rated operating temperature of 605 °C and a rated internal pressure of 27.5 MPa. Detailed specifications are provided in Table 1, indicating that the 1# pipe sample was collected from the boiler plant section, the 2# pipe sample from the front of the high bypass valve, and the 3# pipe sample from the design institute section. Considering the varying specifications of the three pipe sections, the converted internal pressure stress was categorized into three levels, with the 3# pipe sample experiencing the highest stress level, and the 1# pipe sample endured the lowest stress level.

When comparing the internal pressure-converted stress of these samples with the allowable stress at 610 °C, calculated using the interpolation method from different versions of the ASME CC2179 standard [26] for P92 steel as shown in Table 2, several observations are evident. The internal pressure-converted stress of the 1# pipe sample exceeded the allowable stress according to the 2023 version of the standard, yet it remained within the allowable limits specified by the 2006 and 1999 standards. For the 2# pipe sample, the converted internal pressure stress was 78.0 MPa, which was slightly lower than the allowable stress in the 1999 version but not considered safe according to the 2006 and 2023 versions. The 3# pipe sample had the highest stress, surpassing the allowable stress in all three standard versions at 610 °C.

The measured chemical compositions of the three running pipes are shown in Table 3. The chemical composition analysis method was carried out by using the Inductively Coupled Plasma (ICP) method, and each sample was tested three times to take the average value. Most of the elements in the measured P92 steel were close, but the N/Al ratio was quite different. The N/Al ratio was highest in the 3# running pipe, lowest in the 1# running pipe, and the B content in the 3# pipe was marginally higher than in the 1# and 2# pipes.

Despite being sampled from different locations along the main steam pipeline, the steam temperature and pressure exhibited only a slight decrease from the boiler outlet (3#) to the design institute section (1#), and further to the high bypass valve (2#), with the variation being minimal. By comparing and analyzing the evolution of mechanical properties and microstructure over time across the three pipe samples, we can determine the effects of the operational time and stress on the aging characteristics of the material.

## 3. Test Results

### 3.1. Tensile Strength

**Figure 1:**
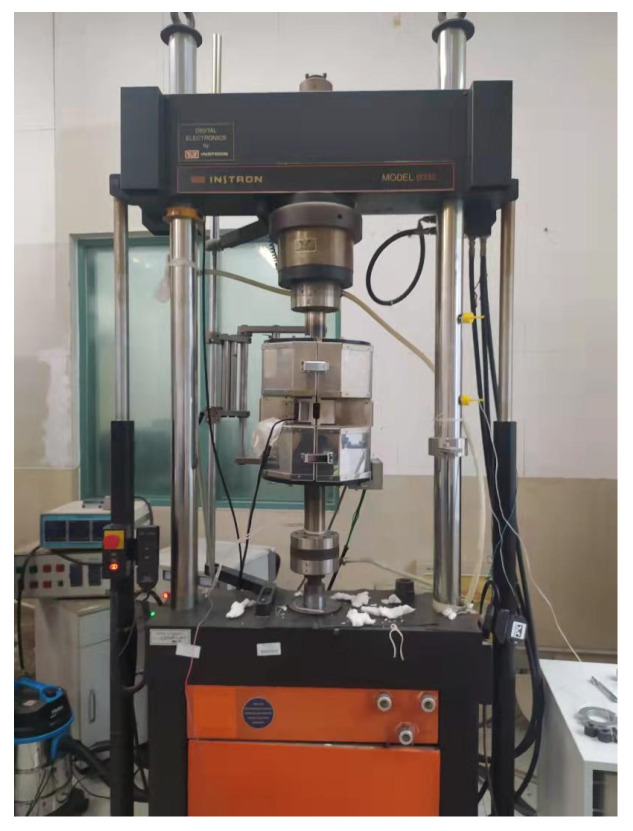
Instron-8032 electro-hydraulic servo universal testing machine.

**Figure 2:**
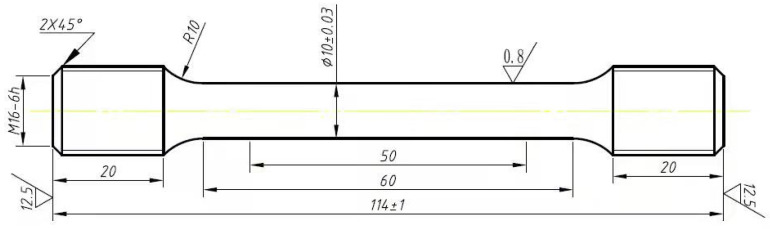
Geometric dimensions of the high-temperature tensile test specimens.

**Figure 3:**
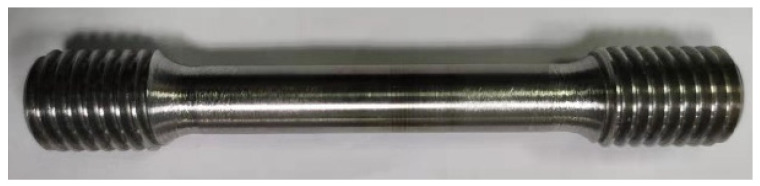
Photographs of the high-temperature tensile test specimens.

**Figure 4:**
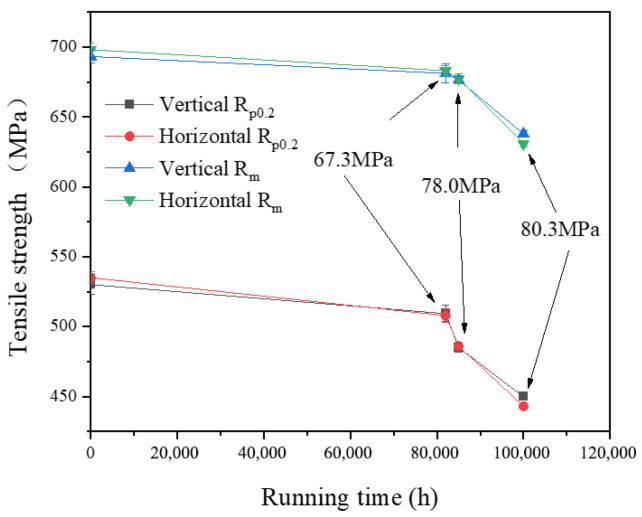
The tensile strength of P92 pipe at RT.

**Figure 5:**
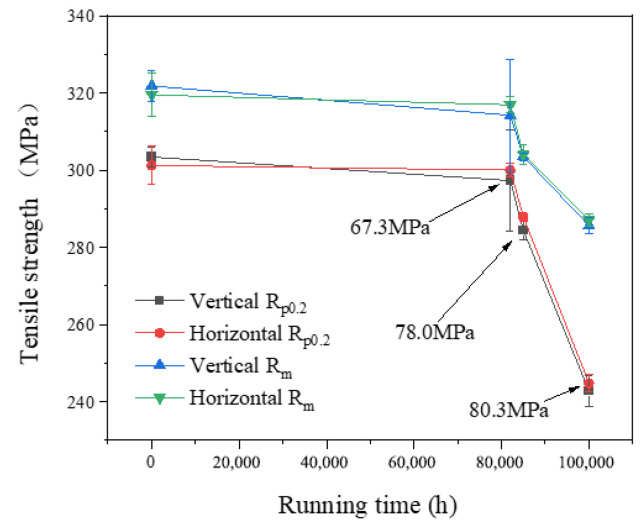
The tensile strength of P92 pipe at 605 °C.

Tensile tests at room temperature and at an elevated temperature of 605 °C were conducted using an Instron-8032 electro-hydraulic servo universal testing machine, as shown in Figure 1. Instron-8032 electro-hydraulic servo universal testing machine., with a maximum load capacity of 100 KN. The mainframe of the electro-hydraulic servo universal testing machine was equipped with a servo actuator that had low damping and high responsiveness. The control system utilized the principle of electro-hydraulic servo closed-loop control, characterized by a fast response, high control precision, a wide frequency range, and a variety of test waveforms. Standard tensile specimens with circular cross-sections were produced for P92 steel pipes with different running times. The tensile test specimens were designed according to GB/T228.1-2015 [27] and ASTM E21 [28] standards. The geometric dimensions of the tensile specimens are depicted in Figure 2, and the physical photographs are presented in Figure 3. Photographs of the high-temperature tensile test specimens. The test temperatures were room temperature and 605 °C, with a tensile strain rate of 0.2 mm/min. After the test temperature was elevated to 605 °C, the specimens were maintained at this temperature for 60 min. The final fracture occurred within the parallel section, confirming the validity of the test results. The sampling positions included the following: near the outer wall, within the midsection of the wall thickness, and near the inner wall, with the sampling orientations extending across both transverse and longitudinal axes. The tensile tests at room temperature and 605 °C were carried out, and the results are shown in Figure 4 and Figure 5. It can be observed that the tensile strength at room temperature and at high temperature were similar across different sampling positions. Furthermore, the variance of data at each stage was minimal, indicating that the data deviation at different positions outside, in, and inside the pipeline was small. The relationship between room-temperature tensile strength and high-temperature tensile strength and running time was largely consistent, both demonstrating a diminishing trend with an increase in operational duration. Notably, the rate of decline became more pronounced between 82,000 h and 100,000 h. Compared with the 1# pipe that had been in operation for 82,000 h, the 3# pipe that had been in operation for 100,000 h showed an increase in the reduction of the longitudinal *R*_p0.2_ at room temperature from 3.9% to 15.1%, the reduction of the transverse *R*_p0.2_ from 5.1% to 17.2%, the reduction of the longitudinal *R*_m_ from 1.7% to 8.0%, and the reduction of the transverse *R*_m_ from 2.2% to 9.7%. At 605 °C, the reduction of the longitudinal *R*_p0.2_ increased from 2.0% to 20%, the reduction of the transverse *R*_p0.2_ increased from 0.4% to 18.7%, the reduction of the longitudinal *R*_m_ at 605 °C increased from 2.4% to 11.2%, and the reduction of the transverse *R*_m_ at 605 °C increased from 0.8% to 10.1%. With the extension of the operating time and the increase in the operating stress, the room-temperature and high-temperature tensile properties of P92 steel deteriorated significantly. The decrease in non-proportional tensile strength (*R*_p0.2_) was obviously greater than that in the tensile strength (*R*_m_).

The observed decrease in the rate of tensile strength between 82,000 h and 100,000 h may have been related to the distinct stress states experienced by the three operational pipes. The circumferential stress of the running pipes increased, which led to the accelerated aging of materials and the increase in the decrease rate of tensile strength of the pipe samples at room temperature and high temperature.

### 3.2. Impact Toughness and Ductile–Brittle Transition Temperature

**Figure 6:**
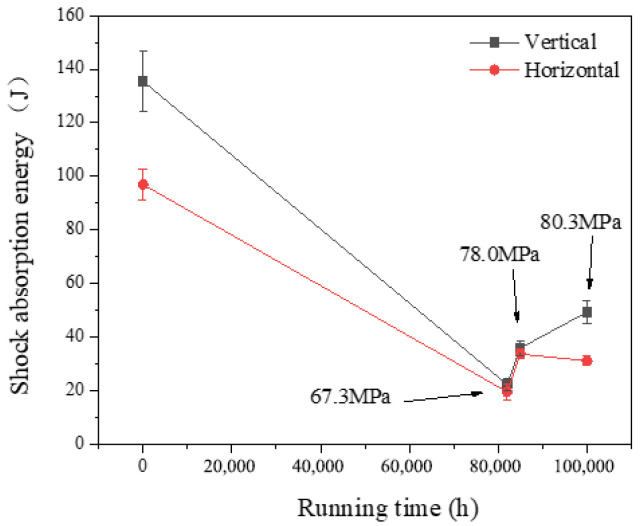
The impact toughness of P92 pipes at room temperature.

**Figure 7:**
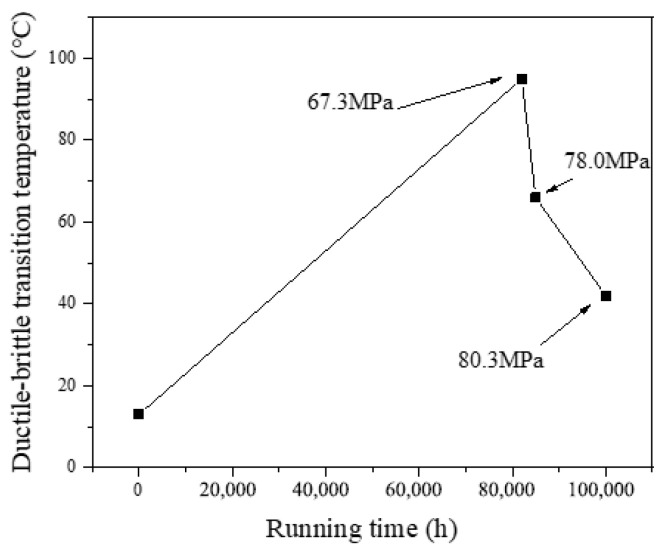
The FATT50 of P92 pipe.

The impact tests were conducted in accordance with the GB/T18658-2002 [29] pendulum impact testing machine inspection standard, measured on a German-made Zwick/Roell 450 impact testing machine, with a maximum impact energy of 300 Joules and a maximum impact velocity of 523 m per second. The specimens used were standard V-notch samples measuring 10 × 10 × 55 mm, and the results are presented in Figure 6 and Figure 7. The impact absorption energy for the transverse samples was observed to be marginally lower compared to the longitudinal samples. Additionally, with an increased operational duration, the impact absorption energy at room temperature underwent a marked decrease initially, followed by a modest elevation. The impact absorption energy of the 1# pipe was the lowest. Compared with the supply state, the reductions in the longitudinal and transverse impact absorption energy were 83.5% and 79.8%, respectively. The impact absorption energy of the 1# pipe was the lowest. Compared with the 0# received pipe, the reductions in impact absorption energy for the longitudinal and transverse directions were 83.5% and 79.8%, respectively. For the 2# pipe, these reductions were slightly less severe, at 73.4% and 65.2% for the longitudinal and transverse directions, respectively. The 3# pipe exhibited even less pronounced decreases, with 63.5% and 67.8% reductions in the longitudinal and transverse impact absorption energies, respectively.

The FATT50 of P92 steel pipe in the supply state was about 13 °C. For the operational pipes, FATT50 was much higher than that in the supply state and significantly higher than at room temperature. However, the relationship between FATT50 and operational duration was non-monotonic, with a peak observed at 95 °C after 82,000 h of operation, subsequently exhibiting a gradual decline to 66 °C and 42 °C as the operational time extended.

According to the existing literature [30,31,32], the impact toughness of T/P92 steel typically shows a sharp decrease in impact absorption energy during the initial aging stage, followed by stabilization. In this study, the impact absorption energy and FATT50 demonstrated a pattern of initially decreasing, then increasing, and finally decreasing again. It is speculated that this phenomenon was related to the circumferential stress differences among the three pipe samples.

### 3.3. Brinell Hardness

**Figure 8:**
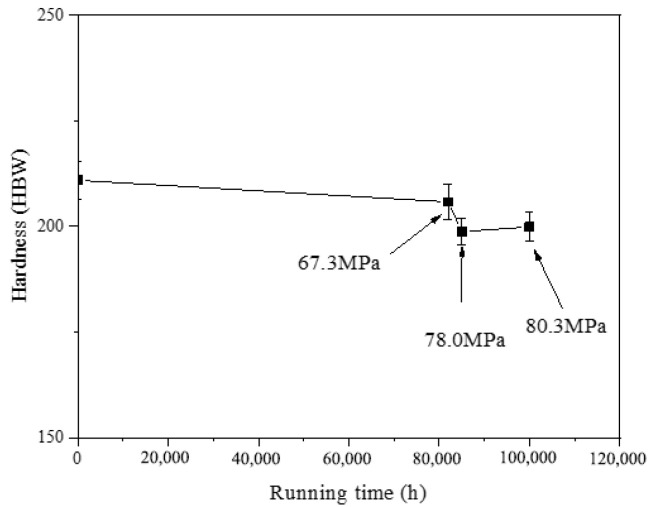
The Brinell hardness variation of P92 pipes.

The Brinell hardness test results for P92 steel pipes after different operational periods are presented in Figure 8. It is evident that the Brinell hardness of P92 steel pipes in operational conditions was consistently lower than those in the supply state. The Brinell hardness reduction of the 1# pipe was only 2.4% compared with that of the 0# pipe, the hardness reduction of the 2# pipe was the largest at 8.2%, and the hardness reduction of the 3# pipe was only 5.2%. With the extension of the operating time, the Brinell hardness of the P92 pipe first decreased and then stabilized, and the change range was not significant. There was no obvious evidence for the influence of the circumferential stress of the pipeline on the Brinell hardness of the material.

The issue of low hardness of T/P92 found in the on-site inspection of thermal power units is often caused by improper post-weld heat treatment or the overtemperature of heating surface tubes. It is uncommon for hardness to decrease abruptly following extended operation in the absence of overheating, a finding that aligns with the results of the hardness tests conducted in this study.

### 3.4. Creep Life Consumption

**Figure 9:**
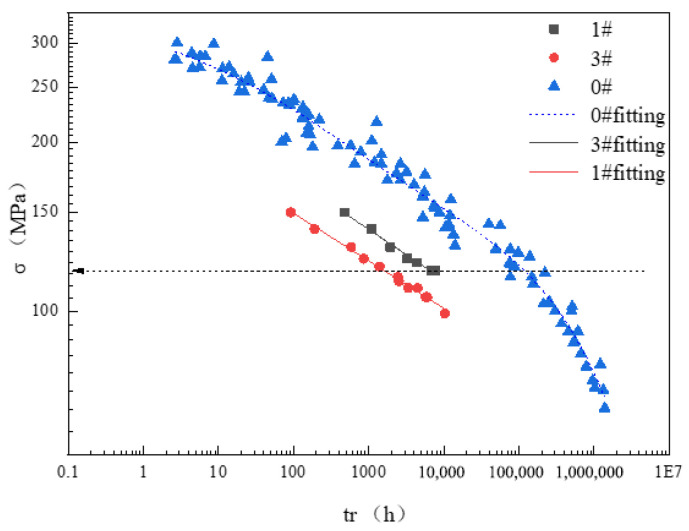
The creep rupture date of P92 pipes at 605 °C.

Figure 9 illustrates the comparative creep endurance data for P92 steel in both supply and operational states at 605 °C. The 0# supply state data, sourced from the U.S. PCC (formerly Weiman Gordon) P92 manual [33], were transformed using the Larson–Miller parameter to determine the equivalent rupture times at 605 °C and the corresponding stress levels for each data point. The data for the 1# and 3# operating tubes represented the measured creep endurance for the transverse samples. It was evident that the creep rupture time of the 1# and 3# operating pipes under identical stress conditions was significantly shorter than that of the 0# supply pipe. Moreover, the creep rupture time of the 3# operating pipe was notably less than that of the 1# operating pipe, indicating a shorter creep residual life for the 3# pipe compared to the 1# pipe. The creep rupture time (*t_r_*) under the same stress conditions was considered the remaining life of the running pipe, while the initial life was defined by the creep rupture time (*t*_0_) of the 0# pipe under specific stress, as illustrated by the dashed horizontal line in Figure 9. The creep life consumption, or creep damage, of the running pipe is calculated as follows:

(1) $$D_{c}=1-t_{r}/t_{0}$$

Without considering the safety factor, by referencing the creep endurance life corresponding to the operating stress of 80 MPa under rated conditions from the chart, we calculated that the creep life consumption for the 1# operating pipe was approximately 42.7%, while for the 3# operating pipe, it amounted to 82.2%.

### 3.5. Microstructure

**Figure 10:**
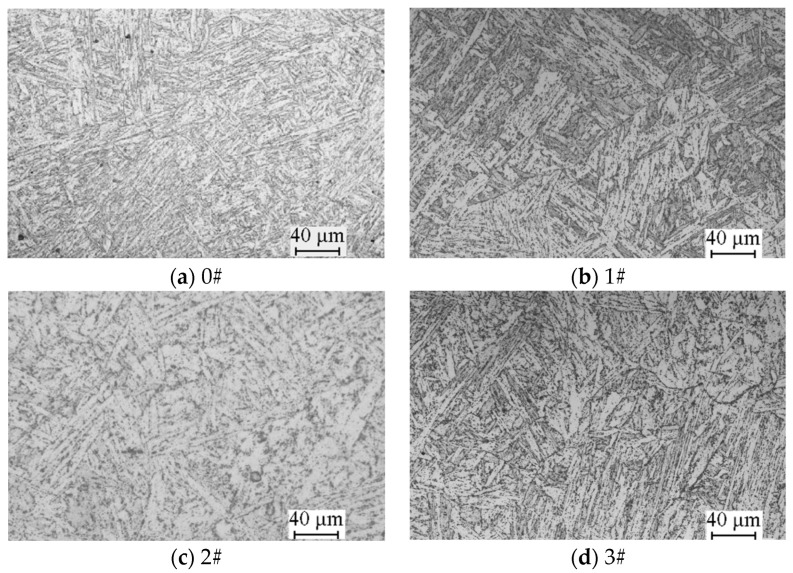
The optical metallographic morphologies of the P92 pipes.

Figure 10 presents optical metallographic images of P92 steel pipe samples under various conditions. It is observable that the martensite lath orientation of the 0# as-received pipe was complete and clear, and the fine second phase was dispersed. In all three operational samples, the martensite lath orientation became dispersed, and the second phase particles exhibited an increase in size. However, no discernible pattern was observed in the optical metallographic images of the 1#, 2#, and 3# operational tubes, despite their significantly differing mechanical properties. This lack of pattern complicated the assessment of the material aging degree based exclusively on these images.

**Figure 11:**
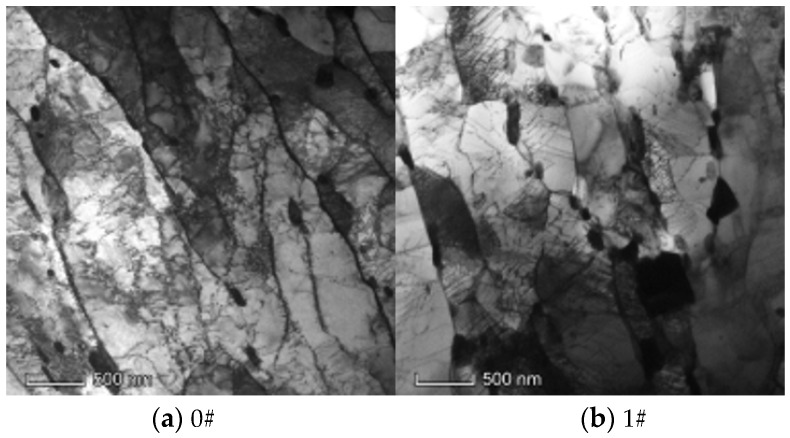

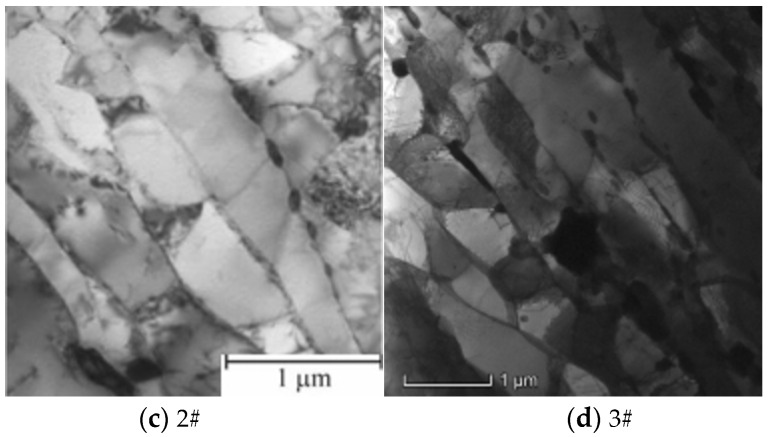
The TEM images of the P92 pipes.

Figure 11 depicts the TEM images of P92 steel pipe samples across different conditions. It is evident that, with a prolonged operation time, the dislocation density decreased markedly, the martensite lath gradually recovered and became segmented by subgrain boundaries, and there was a propensity for the second-phase particles at the lath boundaries to enlarge.

## 4. Analysis and Discussion

The strengthening mechanisms of P92 steel encompass solution strengthening, precipitation strengthening, and dislocation strengthening, among others. After aging, creep, or prolonged operation, the martensitic structure undergoes a reversal. The dislocation density significantly decreases, the lath width increases, and it is gradually subdivided into subgrains by dislocation walls [34,35,36]. During the initial service period, the Laves phase precipitated at the lath boundaries, contributing to precipitation strengthening when its size was small. With prolonged operation, the M_23_C_6_ and Laves phases gradually grew and coarsened, with the Laves phase growing at a faster rate than the M_23_C_6_ phase, leading to clustering phenomena. Due to the high Mo and W content in the Laves phase, its rapid precipitation and growth result in a reduction in solid solution strengthening [37,38]. As the size of the clusters increases, the dislocation pinning effect weakens, leading to a decrease in the strength of P92 steel.

Research indicates that stress can enhance the precipitation and coarsening of M_23_C_6_ phase and the Laves phase of P92 steel [39,40,41,42], but the stress level has different effects on the two main precipitation phases. Scholars [43,44] have discovered that under high-stress conditions, the aggregation and coarsening of M_23_C_6_ phase particles predominate, whereas under low-stress conditions, the aggregation and coarsening of both Laves and M_23_C_6_ phase particles occur in parallel. Additionally, stress can induce the widening of martensite laths and facilitate the transformation from martensite to ferrite [45,46,47]. Two mechanisms account for the widening of martensite laths after long-term service: “Y” lath boundary movement and the merging of parallel lath boundaries. The “Y” lath boundary movement mechanism entails that under the influence of temperature and stress, the “Y” interface of the martensite lath shifts, thereby widening the lath. The merging mechanism of parallel lath boundaries involves movable dislocations within parallel laths consistently migrating to the lath interface to counterbalance dislocations of varying numbers on the interface, leading to the gradual disappearance of the interface between two parallel laths and the subsequent widening of martensite laths. Furthermore, the experimental findings presented in this study, pertaining to the P92 main steam pipeline subjected to long-term service without exceeding temperature limits, suggested that elevated stress levels significantly exacerbated the degradation of the material’s mechanical properties. For instance, the creep life consumption of the 3# operational pipe had reached 82.2%. Near the end of its service life, the mechanical properties of the materials had significantly deteriorated, yet the aging state could not be effectively assessed from its metallographic structure, Brinell hardness, impact energy absorption, and ductile–brittle transition temperature. TEM images can reveal microstructural degradation characteristics such as martensite lath recovery and second phase particle growth, but extensive research and statistical analysis of specific parameter changes are required. This necessitates a high level of expertise from researchers and is challenging to apply in practical engineering contexts.

This study involves the dissection and analysis of a set of T92 internal pressure creep samples, which encompass a complete spectrum of creep life loss from 0% to 100%. The correlation between the mechanical properties and the aging degree of the T92 pipe samples with varying creep levels was examined through tensile testing, hardness testing, and impact testing. Additionally, the evolution of the mechanical properties of T92 during creep was investigated. It was found that room temperature tensile strength, martensite lath width, and the size of secondary phases were closely related to life consumption, potentially serving as indices for evaluating the aging state of T/P92 steel.

**Figure 12:**
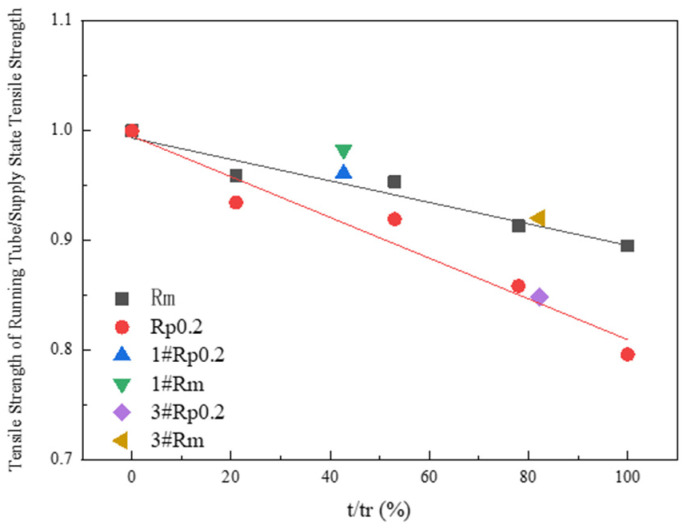
Comparison diagram of life consumption data and corresponding tensile strength decrease in the running pipes of sample 1# and 3# P92 steel.

A comparison of the life consumption data for the 1# and 3# P92 steel pipes was delineated in this study, with the corresponding decreases in tensile strength illustrated in Figure 12. It is evident that the *R*_m_ and *R*_p0.2_ of the 3# pipe sample to the supply state aligned with the relationship of T92 internal pressure creep life consumption. In contrast, the decrease in tensile strength of the 1# pipe sample was significantly less pronounced than that of the internal pressure creep pipe sample at the corresponding creep life consumption. This discrepancy may be attributed to the remaining life of the 1# pipe sample still being considerable.

## 5. Conclusions

This study investigated the mechanical properties and microstructural analysis of P92 operational pipes from the initial batch of USC units in China, which have been operating for different times and with different stresses (0# (as-received condition), 1# (82,000 h, 67.3 MPa), 2# (85,000 h, 78.0 MPa), and 3# (100,000 h, 80.3 MPa)). The findings yielded the following conclusions:

1. The escalation of internal pressure during operation leads to a substantial rise in circumferential stress, which in turn markedly accelerates the degradation of tensile properties in operational pipes. This phenomenon significantly expedites the depletion of the creep life of P92 steel. In comparison to its as-supplied condition, the 3# pipe, which had experienced 82.2% creep life consumption, exhibits a 17.2% decrease in room temperature yield strength (*R*_p0.2_) and a 9.7% decrease in tensile strength (*R*_m_). At an elevated temperature of 605 °C, the *R*_p0.2_ diminishes by 20%, while the tensile strength (*R*_m_) decreases by 11.2%. The observed reduction in *R*_p0.2_ is notably more pronounced than that of *R*_m_. As the operational duration and stress levels escalate, the tensile properties of P92 steel at both room temperature and high temperatures exhibit a significant decline. The *R*_p0.2_ and *R*_m_ of the P92 steel main steam pipeline at both room temperature and 605 °C high temperature exhibit a monotonic decline with increasing service time. This decline is indicative of the stress influence and holds potential for application in the engineering assessment of the residual life of P92 steel.
2. The impact absorption energy, FATT50, and Brinell hardness of the P92 operating pipes do not exhibit a direct correlation with operational time. Specifically, the 1# pipe demonstrates the lowest impact absorption energy among the samples tested. In comparison to their as-supplied state, the P92 steel operating pipes exhibit significant decreases in impact absorption energy in both longitudinal and transverse directions. Specifically, the reductions are 83.5% and 79.8% for the longitudinal and transverse impacts, respectively. For the 2# pipe, these reductions are slightly less pronounced, with 73.4% and 65.2% decreases for longitudinal and transverse impacts, respectively. The 3# pipe shows further attenuation, with reductions of 63.5% and 67.8% for longitudinal and transverse impacts, respectively. The FATT50 value of P92 steel reaches its peak at 95 °C after 82,000 h of operation and subsequently declines to 66 °C and 42 °C with the prolongation of service time and the increase in stress levels. Concurrently, the Brinell hardness of the P92 steel pipes post-operation registers lower values compared to the as-supplied state. Among them, the 1# pipe exhibits the least reduction in hardness at 2.4%, while the 2# pipe experiences the most significant decrease at 8.2%, and the 3# pipe shows a moderate reduction of 5.2%. These findings suggest that the impact toughness, FATT50, and Brinell hardness of P92 steel are not sensitive to the aging of the material caused by stress.
3. There is no obvious regular difference in the optical metallographic photos of the three P92 operating pipes. It is difficult to judge the degree of performance degradation caused by the increase in stress only from the optical metallographic photos, and the aging degree of its microstructure needs to be observed by TEM.

## Figures and Tables

**Table 1:** Sample information.

Specimen Number	Running Time/Kh	Specification/mm	Internal Pressure Conversion Stress/MPa
0#	0	ID 248 × 53	/
1#	8.2	Φ559 × 95	67.3
2#	8.5	ID 248 × 53	78.0
3#	10	ID349 × 72	80.3

**Table 2:** Allowable stress of P92 pipe in different version standards.

Vintages	Standard Number	Permissible Stress at 610 °C/MPa
1999	CC2179-3	79.4
2006	CC2179-6	68.8
2023	CC2179-11	66.7

**Table 3:** Chemical composition of P92 pipe (wt. %).

Elemental	1#	2#	3#	Elemental	1#	2#	3#
C	0.096	0.10	0.095	V	0.21	0.16	0.18
Si	0.42	0.37	0.29	Ti	<0.005	0.0025	<0.005
Mn	0.47	0.47	0.47	Nb	0.058	0.061	0.057
P	0.011	0.017	0.013	Al	0.03	0.0075	0.0053
S	0.001	0.0088	0.014	B	0.0025	0.0014	0.0055
Cr	8.68	8.50	8.88	W	1.77	1.85	1.68
Ni	0.25	0.40	0.37	Zr	<0.01	0.0082	<0.005
Mo	0.37	0.39	0.38	N	0.039	0.048	0.048
Cu	0.16	0.15	0.16	N/Al	1.3	6.4	9.1

## Data Availability

The data presented in this study are available on request from the corresponding author.
